# Long-term trends and future projections of the burden of tuberculosis among children and adolescents in China

**DOI:** 10.1371/journal.pone.0328255

**Published:** 2025-07-17

**Authors:** Nan Li, Wei Wei, Yinglin Lao, Xi Zhu, Qi Ye, Junhong Chen, Yating Ji, Ruoqing Chen, Chongguang Yang

**Affiliations:** 1 School of Public Health (Shenzhen), Shenzhen Campus of Sun Yat-sen University, Shenzhen, Guangdong, China; 2 School of Public Health (Shenzhen), Sun Yat-sen University, Shenzhen, Guangdong, China; 3 Institute of Environmental Medicine, Karolinska Institutet, Stockholm, Sweden; 4 School of Public Health (Shenzhen), Shenzhen Key Laboratory of Pathogenic Microbes and Biosafety, Shenzhen Campus of Sun Yat-sen University, Sun Yat-sen University, Guangzhou, Guangdong, China; 5 Guangdong Provincial Highly Pathogenic Microorganism Science Data Center, Key Laboratory of Tropical Disease Control, Ministry of Education, Sun Yat-sen University, Guangzhou, Guangdong, China; Atal Bihari Vajpayee Institute of Medical Sciences & Dr Ram Manohar Lohia Hospital, INDIA

## Abstract

**Background:**

China ranks third in estimated TB incidence in 2023, accounting for 6.8% of the global cases. TB in children and adolescents is a public health issue that today warrants priority attention in China.

**Objective:**

The purpose of this study was to investigate the burden of TB among Chinese children and adolescents aged 0–19 years from 1990 to 2021 and to estimate the incidence rate, mortality rate, and disability-adjusted life years (DALYs) rate from 2022 to 2031.

**Methods:**

The Joinpoint regression analysis was used to identify periods of significant change and autoregressive Integrated Moving Average (ARIMA) modeling was employed to predict the TB burden for 2022–2031.

**Results:**

The study indicated that China has significantly reduced the TB burden among children and adolescents over the past 32 years, the most pronounced reductions in incidence occurred during the periods 2010–2015 (APC = −8.64%, P < 0.05) and 2019–2021 (APC = −6.09%, P < 0.05). Meanwhile, death and DALYs rates showed a consistently rapid decline across the entire 32-year span. Adolescents aged 15–19 years have the highest incidence rates, and children under 5 continue to face high mortality and DALYs rates. Additionally, females experienced a more significant decline compared to males across all age groups. Despite minor fluctuations in some age groups, a downward trend in incidence, death, and DALYs rates was anticipated to continue until 2031, with persistent gender differences in future projections.

**Conclusions:**

Our findings demonstrate a persistent downward trajectory in TB burden among Chinese children and adolescents from 1990 to 2021, with significant gender disparities favoring females across all age groups. Notably, children younger than 5 years and adolescents aged 15–19 years are at higher risk, which emphasizes the importance of tailored interventions to ensure continued progress towards comprehensive TB control goals.

## Introduction

The impact of TB on children and adolescents is a significant global health concern. According to estimates from the World Health Organization (WHO), approximately 1.25 million children aged 0–14 years were affected by TB in 2022, accounting for 12% of all TB cases. Furthermore, around 214,000 children are expected to die from TB, representing 16% of all TB-related deaths [1]. This issue is equally pressing in China. In 2021, the adolescent population aged 10–19 years was estimated to exceed 162 million, making up 11.49% of the total population [2]. Most of these individuals are students, and in the same year, approximately 39,900 TB cases were reported in schools across the country [3].

While TB incidence and prevalence decline markedly during mid-childhood (ages 5–9 years) compared to early childhood (<5 years), they rise significantly during adolescence (ages 10–19 years) [4–7]. Adolescence is characterized by unique biological and social changes, including hormonal and immunological shifts, increasing independence, intensified social interactions, and heightened risk-taking behaviors [8]. As the incidence of TB increases among adolescents, so too does the need for improved identification, treatment, and control efforts. Indeed, adolescent TB control has become a key focus of the WHO’s global roadmap to TB elimination [1]. Despite progress in global TB treatment, the number of children receiving care remains insufficient. Between 2018 and 2022, an estimated 2.5 million children received treatment, representing only 71% of the 3.5 million target set by the United Nations’ 2018 High-Level Meeting [9], underscoring the ongoing challenges in meeting global goals.

China is one of the 30 high-TB burden countries, ranking third globally in terms of total TB cases, accounting for 6.8% of all cases worldwide [10]. However, the full extent of TB burden among children and adolescents in China remains inadequately understood. A comprehensive assessment of TB burden in this age group is critical to developing targeted interventions and optimizing resource allocation. This study aims to examine the burden of TB among children and adolescents in China from 1990 to 2021 and to predict trends from 2022 to 2031. The findings will provide a solid evidence base to inform public health policy, improve resource distribution, and design effective strategies to mitigate the impact of TB on children and adolescents in China.

## Methods

### Data sources

The data for this study were sourced from the Global Burden of Disease Study 2021 (GBD 2021) Results, produced by the Institute for Health Metrics and Evaluation (IHME) in Seattle, United States (2022). The data were accessed through the GBD Results Tool (https://vizhub.healthdata.org/gbd-results/). The GBD 2021 database integrated various data sources, including national censuses, household surveys, disease monitoring systems, and other relevant data repositories. The database covers 371 diseases and injuries, as well as 88 risk factors, across 204 countries and territories, spanning the period from 1990 to 2021. In this study, we specifically retrieved data on TB incidence, death, and disability-adjusted life years (DALYs) for Chinese children and adolescents aged 0–19 years.

### Definitions

In this study, DALYs for TB were defined as the sum of years of life lost (YLL) and years lived with disability (YLD) attributable to tuberculosis in the specified population. The DALYs rate was calculated as the number of DALYs per 100,000 population. Incidence and death were defined as the number of new cases and deaths per 100,000 population, respectively. For each of these measures, we report 95% uncertainty intervals (UIs), which were derived by sampling 1000 values from the posterior distribution of each estimate. The 2.5th and 97.5th percentiles of these samples were used to calculate the uncertainty intervals, reflecting the statistical uncertainty inherent in the data.

### Statistical analysis

To gain a deeper understanding of the burden of TB among children and adolescents in China, we analyzed trends in TB incidence, DALYs and death rates for the population aged 0–19 years from 1990 to 2021. Joinpoint regression models were employed to assess the temporal trends in TB-related incidence, death, and DALYs. To quantify the rate of change in these indicators, we used Log-linear regression models to calculate the annual percentage change (APC) and the average annual percentage change (AAPC) along with 95% uncertainty intervals (UIs) by age and gender. If the APC/AAPC values and the lower 95% CI were both > 0, the indicators were considered to be increasing; if the APC/AAPC values and the upper 95% CI were both < 0, the indicators were considered to be decreasing. The Joinpoint software (version 4.9.1.0; National Cancer Institute, Rockville, MD, USA) was used to perform the regression analysis. Autoregressive integrated moving average (ARIMA) models, which combine autoregressive (AR) and moving average (MA) components with differencing (d) to stabilize the series, were used to predict these indicators from 2022 to 2031. In the ARIMA (p, d, q) model, “p” denotes the count of autoregressive terms, “d” represents the order of differencing, and “q” indicates the number of moving average terms. The ARIMA model was trained on TB incidence, death, and DALYs data from 1990 to 2021. To select the most suitable model for the time series data, we scrutinized the autocorrelation function (ACF) and partial autocorrelation function (PACF) plots, and also considered the Ljung-Box test results. These analyses allowed us to evaluate the temporal patterns and the degree of smoothness inherent in the data [11]. The best-fitting ARIMA model was selected based on these diagnostic checks. P-value < 0.05 was considered statistically significant.

## Results

### Overview

**Fig 1:**
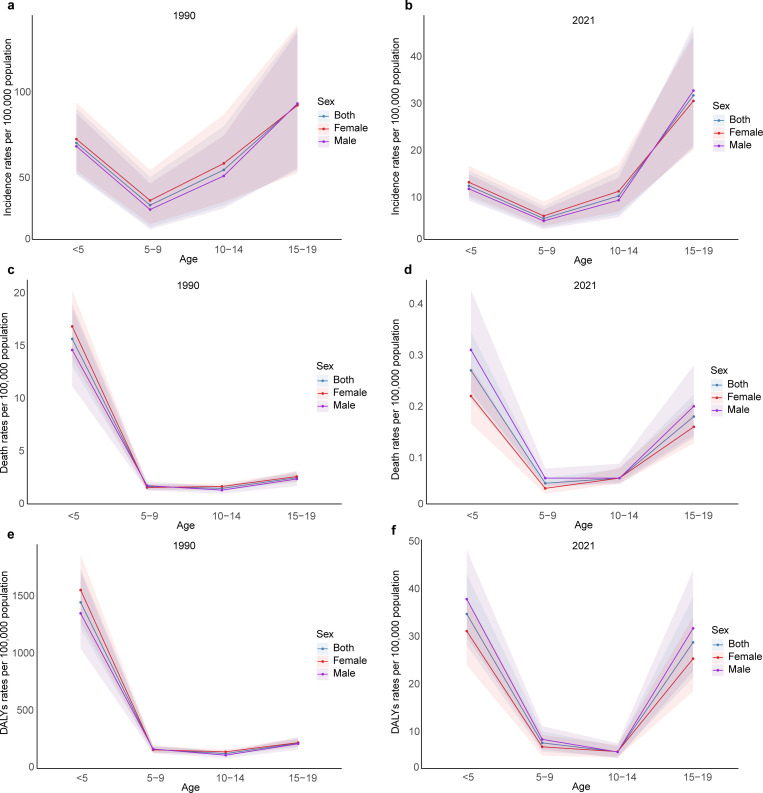
Tuberculosis incidence rates, death rates, and Disability-Adjusted Life Years (DALYs) rates Across Different Age Groups in 1990 and 2021. a-b depicts the incidence rates for different age groups in the years 1990 and 2021, c-d represents the death rates, and e-f denotes the DALYs rates. The shaded area represents the 95% uncertainty interval (UI).

In 2021, among children and adolescents aged 0–19 years in China, there were 47,616 (95% UI 36,193–60,318) tuberculosis incident cases, 445 (368–540) deaths and 61,277 (50,945–75,534) DALYs due to tuberculosis in China. The incidence, death, and DALYs among children and adolescents in China experienced a significant decrease from 1990 onwards. Specifically, the incidence in 2021 was 14.24 (95% UI 10.83–18.04) per 100,000 population, a marked decrease from 64.64 (95% UI 49.48–82.82) in 1990. The death rate dropped from 5.35 (95% UI 4.57–6.25) per 100,000 population in 1990 to 0.13 (95% UI 0.11–0.16) per 100,000 population in 2021, and DALYs decreased from 485.25 (95% UI 415.28–562.50) per 100,000 population in 1990 to 18.33 (95% UI 15.24–22.59) per 100,000 population in 2021(Table 1). Data from 2021 indicated that the highest incidence rates were observed in individuals aged 15–19 years, with a rate of 31.75 (95% UI 20.64–45.39) per 100,000 population. This was followed by children younger than 5 years, who had an incidence rate of 12.51 (95% UI 9.87–15.77) per 100,000 population. In contrast, the lowest incidence was recorded among those aged 5–9 years, at a rate of 5.53 (95% UI 3.63–8.18) per 100,000 population. Among individuals aged 15–19 years, the incidence was higher in males than in females, while in other age groups, females had a slightly higher incidence than males. The highest death rates were observed in children younger than 5 years, with a rate of 0.27 (95% UI 0.21–0.34) per 100,000 population, This was followed by those aged 15–19 years, who had a death rate of 0.18 (95% UI 0.14–0.22) per 100,000 population. In all age groups, male death rates were higher than female, except for those aged 10–14 years, where death rates were equal. For DALYs, the highest burden was observed in the children younger than 5 years, with a rate of 34.88 (95% UI 28.31–43.22) per 100,000 population. This was followed by those aged 15–19 years, who had a DALYs rate of 28.92 (95% UI 21.55–38.46) per 100,000 population. (S1–S3 Tables, Fig 1). In all age groups, DALYs for males were higher than those for females, except for those aged 10–14 years, where DALYs for females were higher.

**Table 1:** Gender-specific incidence, death, and DALYs rates and their average annual percentage changes (AAPC) from 1990 to 2021 in China.

	Gender	1990	2021	AAPC%(1990–2021)	P
Incidence rates per 100,000 population	Male	62.82 (48.44,79.82)	13.98 (10.56,17.77)	−4.72(−4.88,-4.56)	<0.001
	Female	66.62 (50.45,86.42)	14.54 (11.15,18.58)	−4.79(−4.94,-4.63)	<0.001
	Both	64.64 (49.48,82.82)	14.24 (10.83,18.04)	−4.75(−4.93,-4.58)	<0.001
Deaths rates per 100,000 population	Male	5.1 (3.92,6.36)	0.15 (0.12,0.2)	−10.74(−11.09,-10.40)	<0.001
	Female	5.63 (4.79,6.58)	0.11 (0.09,0.14)	−11.87(−12.25,-11.49)	<0.001
	Both	5.35 (4.57,6.25)	0.13 (0.11,0.16)	−11.27(−11.59,-10.95)	<0.001
DALYs rates per 100,000 population	Male	463.05 (359.34,573.44)	19.96 (16.11,25.12)	−9.65(−10.05,-9.24)	<0.001
	Female	509.27 (432.31,592.32)	16.46 (13.36,19.82)	−10.44(−10.84,-10.04)	<0.001
	Both	485.25 (415.28,562.50)	18.33 (15.24,22.59)	−10.02(−10.40,-9.63)	<0.001

DALYs = disability-adjusted life years; AAPC = Annualised rate of change in tuberculosis incidence, deaths and DALYs; GBD = Global Burden of Diseases, Injuries, and Risk Factors Study. Data in parentheses are 95% uncertainty intervals.

### APC and AAPC

**Fig 2:**
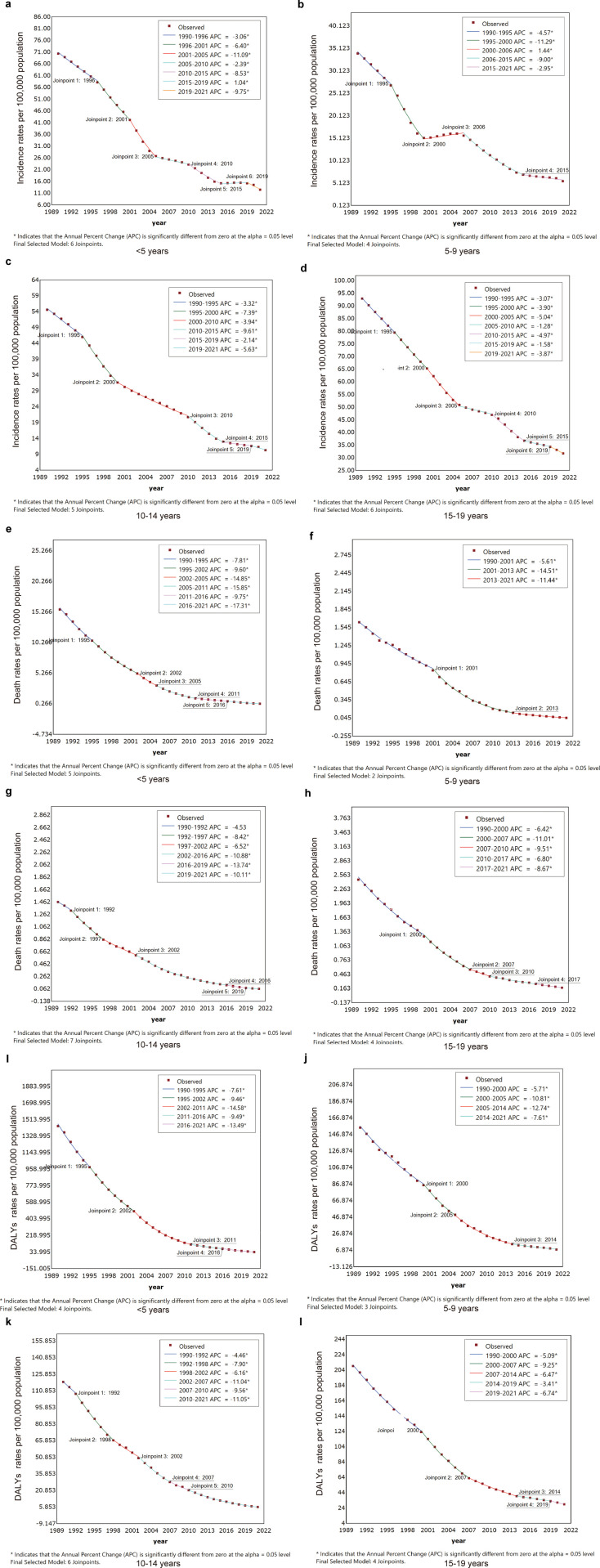
Joinpoint regression results for incidence rates, death rates, and DALYs rates across different age groups. a-d represents the incidence rates across different age groups, e-h denotes the death rates, and i-l indicates the DALYs rates.

From 1990 to 2021, there was a substantial decline in the incidence, death, and DALYs associated with tuberculosis (TB) among children and adolescents aged 0–19 years in China. Over these 32 years, the average annual percent changes (AAPC) for TB incidence, death, and DALYs rates were −4.75% (95% UI −4.93 to −4.58), −11.27% (95% UI −11.59 to −10.95), and −10.02% (95% UI −10.40 to −9.63), respectively. The most pronounced reductions in incidence occurred during the periods 2010–2015 (APC = −8.64%, P < 0.05) and 2019–2021 (APC = −6.09%, P < 0.05). Meanwhile, death and DALYs rates showed a consistently rapid decline across the entire 32-year span (Table 1, S1 Fig). The most substantial decline in the incidence of tuberculosis among those younger than 5 years occurred between 2001 and 2005 (APC = −11.09%, *P* < 0.05), followed by another notable reduction from 2019 to 2021 (APC = −9.75%, *P* < 0.05). However, an upward trend was observed from 2015 to 2019 (APC = 1.04%, *P* < 0.05) in this group, among both male and female children (APC = 1.04% and 1.02%, respectively, *P* < 0.05). In those aged 5–9 years, the most significant decline occurred from 1995 to 2000 (APC = −11.29%, *P *< 0.05), followed by a brief increase between 2000 and 2006 (APC = 1.44%, *P *< 0.05), with both males and females experiencing a notable rise (APC = 2.60% and 1.06%, respectively, *P* < 0.05). For those aged 10–14 years, the sharpest decline was observed during 2010–2015 (APC = −9.61%, *P *< 0.05), while those aged 15–19 years exhibited a steady decline throughout the study period (S1 Table, Fig 2, S2 Fig). The decline in both death rates and DALYs rates was more pronounced in females than in males across all age groups (S2 and S3 Tables, Fig 2, S3 and S4 Figs).

### Trend analysis and prediction

**Fig 3:**
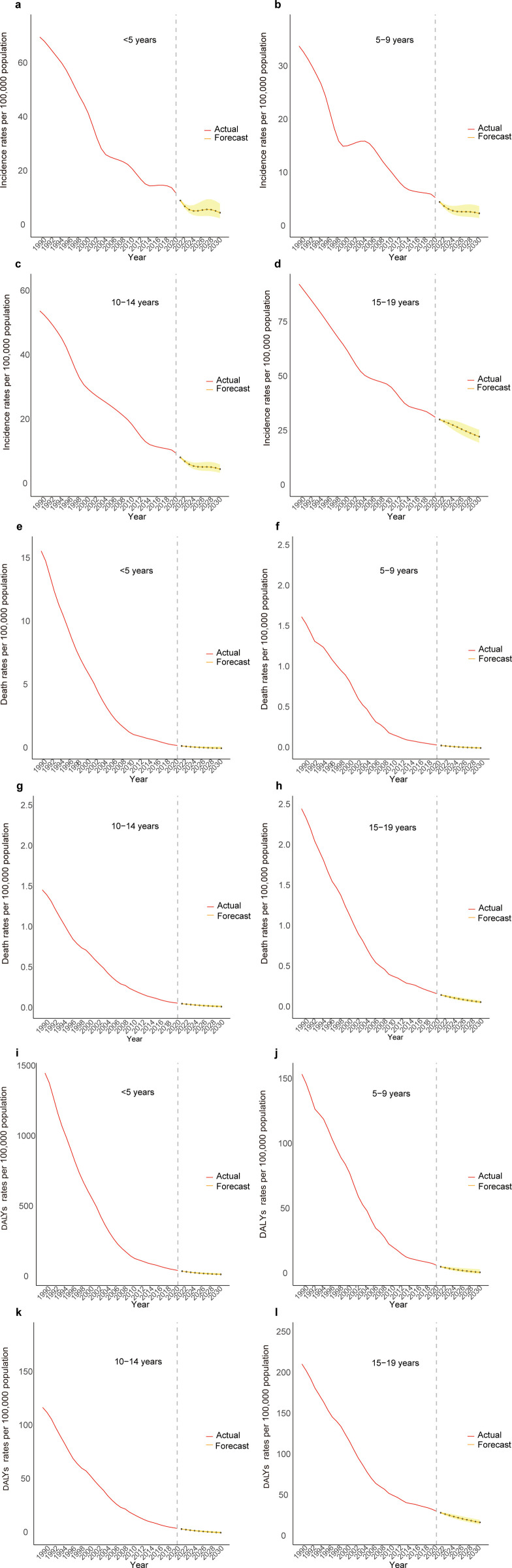
ARIMA prediction results for incidence rates, death rates, and DALYs rates across different age groups. a-d represents ARIMA prediction results for incidence, e-h for death rates, and i-l for DALYs rates. The shaded area represents the 95% uncertainty interval (UI).

**Fig 4:**
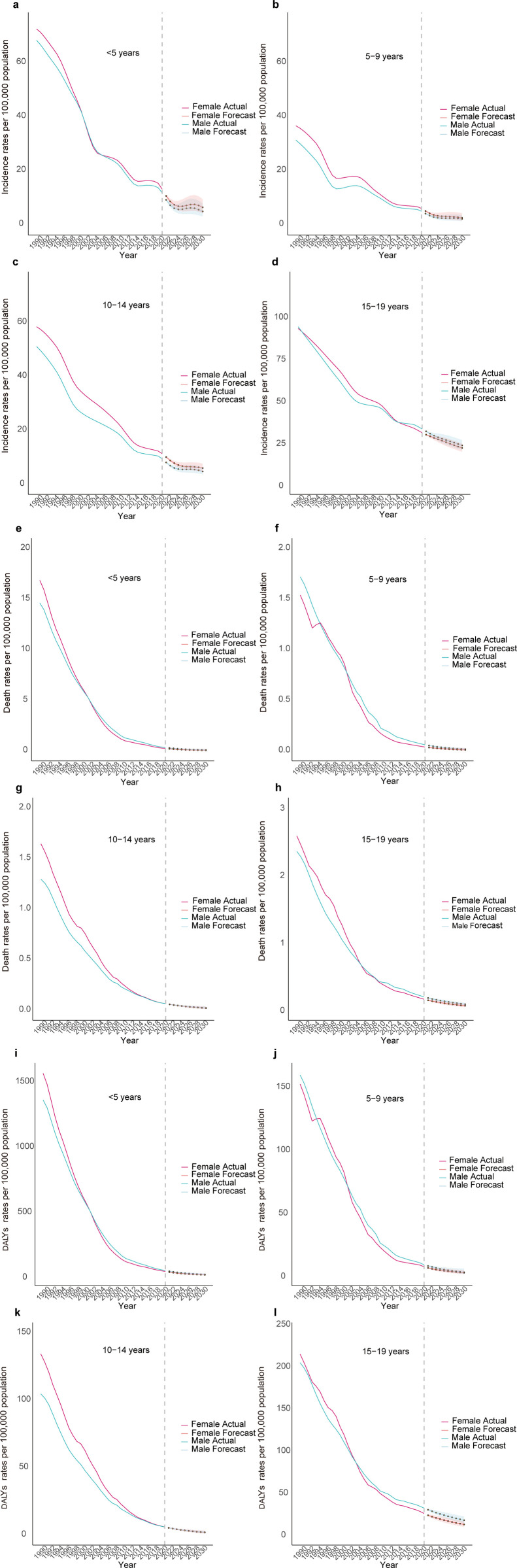
ARIMA prediction results for incidence rates, death rates, and DALYs rates across different age groups and genders. a-d represents the incidence results, e-h denotes the deaths results, and i-l indicates the DALYs results. The shaded area represents the 95% uncertainty interval (UI).

Our estimation for the next decade indicated a general decline in tuberculosis (TB) burden among Chinese children and adolescents, with specific fluctuations. Notably, a temporary increase in incidence rates was expected for those younger than 5 years from 2025 to 2028, followed by a decline, and a similar pattern was projected for those aged 10–14 years in 2027–2028. (Fig 3, S4 Table). Gender-specific analysis revealed that females had higher incidence rates for those younger than 5 years, aged 5–9 years and 10–14 years, while males had exceeded in the 15–19 age group by 2031. Additionally, death and DALYs rates were consistently higher in males across all age groups by 2031. (Fig 4, S4 Table). (See S5 Table for AIC values and Ljung-Box test results of the ARIMA model suggested for incidence, death, and DALYs rates.)

## Discussion

From 1990 to 2021, a significant reduction in tuberculosis (TB) incidence, mortality, and DALYs among Chinese children and adolescents (0–19 years) was observed, reflecting substantial public health progress.

The significant progress made in reducing the TB burden among children and adolescents in China can be attributed to the implementation of public health policies, medical advancements, and China’s socioeconomic development. Notably, increased BCG vaccination coverage has been crucial in reducing childhood TB, despite variable effectiveness against adult TB [12,13]. The nationwide expansion of the Directly Observed Treatment, Short-course (DOTS) strategy in 2005 has improved treatment adherence and success rates, emphasizing the supervision of treatment and community involvement [14]. The 2004 revision of the Infectious Disease Prevention and Control Law and the establishment of the Tuberculosis Information Management System (TBIMS) in 2005 have bolstered TB surveillance and control [15]. These measures, along with improvements in living standards, have collectively reduced the TB burden among children and adolescents [16–19]. The periods 2010–2015 and 2019–2021 saw more pronounced declines in TB incidence. The former can be attributed to the long-term effects of key interventions, including the nationwide DOTS implementation, the 2006 National Guidelines for Childhood TB, and the 2008 National TB Strategy [20,21]. The significant decline in 2019–2021 is complex, with COVID-19 public health measures likely reducing TB transmission but also disrupting health services, potentially leading to under-diagnosis and under-reporting of TB cases [22–25].In conclusion, while the decline in TB burden among children and adolescents in China is commendable, the pandemic’s impact on health services underscores the need for sustained TB surveillance and control. Continued efforts in vaccination, early diagnosis, treatment adherence, and healthcare infrastructure are vital to further reduce TB in this vulnerable demographic.

Analysis of tuberculosis (TB) burden from 1990 to 2021 revealed that adolescents aged 15–19 years had the highest incidence, while children younger than 5 years experienced the highest mortality and DALYs. This aligns with global trends, suggesting increased TB susceptibility in adolescents due to waning BCG vaccine protection and behavioral factors [8,26]. Adolescents’ social interactions and biological changes during this period may heighten TB risk [27–29]. Studies, such as one in Kenya showing a six-fold higher TB prevalence in adolescents than case notifications [30], and research in Botswana indicating higher missed appointment rates for TB treatment in adolescents [31], underscore the need for targeted TB control in this demographic. The vulnerability of children younger than 5 years is attributed to their immature immune systems, leading to rapid disease progression and high fatality rates [32,33]. Diagnostic challenges, including non-specific symptoms and difficulty in obtaining sputum samples, contribute to treatment delays and increased mortality in this age group [34–36]. Enhancing TB control efforts for both age groups involves raising awareness, promoting healthier lifestyles, improving health services, and bolstering early diagnosis and treatment. The period of 2015–2019 saw an increase in TB incidence in children younger than 5 years, possibly due to improved diagnostic capacity and the expansion of China’s national TB control program, facilitating earlier detection, especially in remote areas [37,38].

From 1990 to 2021, gender differences in tuberculosis (TB) incidence among Chinese children and adolescents were pronounced, with higher rates in females, reversing in those aged 15–19 years post-2016. Mortality rates initially favored females but shifted to males by 2002 for those younger than 5 years and by 2006 for those aged 15–19 years. Notably, the rate of decline in female mortality was more pronounced than that of males across all age groups, suggesting a positive impact of recent public health measures on female health outcomes. The observed gender differences in TB incidence and mortality can be attributed to a combination of biological, behavioral, and sociocultural factors. From a biological perspective, sex hormones are known to modulate immune responses, with evidence suggesting that women generally exhibit a stronger immune response to Mycobacterium tuberculosis than men. This enhanced immune activity may confer protection against initial infection but could also increase the risk of progression to active disease in women after infection [39]. Furthermore, differences in metabolism (e.g., iron and lipid processing), genetics (e.g., X-linked genes), and respiratory system anatomy may contribute to the observed gender variations in TB burden. Social and behavioral influences, such as healthcare-seeking behaviors and cultural norms around masculinity, also play a role, with males showing delayed medical attention and higher rates of unhealthy habits like smoking and alcohol use, potentially increasing TB susceptibility [40]. The shift in TB burden towards males, particularly adolescents, calls for gender-sensitive TB control strategies. As TB epidemiology evolves, public health interventions must adapt to address emerging disparities and ensure equitable healthcare for all children and adolescents.

Our analysis reveals significant gender differences in TB burden among Chinese children and adolescents over the next decade. Mortality was highest in children younger than 5 years from 1990 to 2021, but by 2031, adolescents aged 15–19 years are projected to have the highest mortality rates. This shift underscores the need to prioritize adolescent health in TB control strategies [8], Adolescents face challenges in healthcare access, particularly during the transition to adult services, which is exacerbated in TB-endemic regions with limited specialized services for this demographic [28]. Gender differences in disease burden are pronounced, with females projected to have higher incidence rates in younger age groups, while males are expected to surpass those aged 15–19 years. These findings highlight the need for further investigation into gender-specific TB disparities and the development of targeted interventions.

However, the study has limitations. The GBD 2021 methodology, despite its value, may impact the precision of model estimates due to several factors. Firstly, data quality issues have the potential to introduce bias into the analysis. Secondly, the reliance on model-fitting techniques rather than direct case data could result in either overestimating or underestimating the true burden of tuberculosis. These limitations highlight the need for caution when interpreting the results and suggest that future studies might benefit from incorporating more direct epidemiological data to enhance the accuracy of TB burden estimates. The analysis did not consider broader socio-economic impacts or latent TB infection (LTBI) cases, which could affect the accuracy of the results. Future studies should employ a multidimensional approach to enhance the robustness of the findings.

## Conclusions

Despite the remarkable progress China has made in the prevention and control of TB in children and adolescents, as evidenced by marked declines in incidence, mortality, and DALYs, gender and age-related disparities in the burden of TB remain. This study emphasizes the ongoing need for targeted, age- and gender-specific interventions. In particular, adolescents aged 15–19 years, who have the highest incidence rates, and children younger than 5 years, who continue to face high rates of mortality and DALYs, should be prioritized in future TB control strategies. A more comprehensive approach that considers these demographic variations will be essential to further reducing the TB burden in these vulnerable groups.

## Supporting information

S1 Fig Joinpoint regression results for incidence rates, death rates, and DALYs rates in age group 0–19 years, stratified by gender. a-c shows joinpoint results of incidence rates, d-f shows joinpoint results of death rates, and g-i shows joinpoint results of DALYs rates.
(PDF)

S2 Fig Joinpoint regression results for incidence rates across different age groups and genders. a-d shows joinpoint results of male incidence rates in different age groups, and e-h shows joinpoint results of female incidence rates.
(PDF)

S3 Fig Joinpoint regression results for death rates across different age groups and genders. a-d shows joinpoint results of male death rates in different age groups, and e-h shows joinpoint results of female death rates.
(PDF)

S4 Fig Joinpoint regression results for DALYs rates across different age groups and genders. a-d shows joinpoint results of male DALYs rates in different age groups, and e-h shows joinpoint results of female DALYs rates.
(PDF)

S1 Table Gender-specific and age-specific incidence rates and their average annual percentage changes (AAPC) from 1990 to 2021 in China. (PDF)

S2 Table Gender-specific and age-specific death rates and their average annual percentage changes (AAPC) 1990–2021 in China. (PDF)

S3 Table Gender-specific and age-specific DALYs rates and their average annual percentage changes (AAPC) from 1990 to 2021 in China. (PDF)

S4 Table Predicted values of incidence, deaths and DALYs rates in children and adolescents of different ages and genders during 2022–2031. (PDF)

S5 Table AIC values for suggested ARIMA models and Ljung-Box test results for incidence, deaths and DALYs rates of different age groups. (PDF)
