# Supplementary material for: Long-term trends and future projections of the burden of tuberculosis among children and adolescents in China
Source: PLoS One. 2025 Jul 17;20(7):e0328255. doi: 10.1371/journal.pone.0328255 (PMC12270101; doi:10.1371/journal.pone.0328255)
Supplement: S1 Fig — a-c shows joinpoint results of incidence rates, d-f shows joinpoint results of death rates, and g-i shows joinpoint results of DALYs rates. (PDF) [file pone.0328255.s001.pdf]

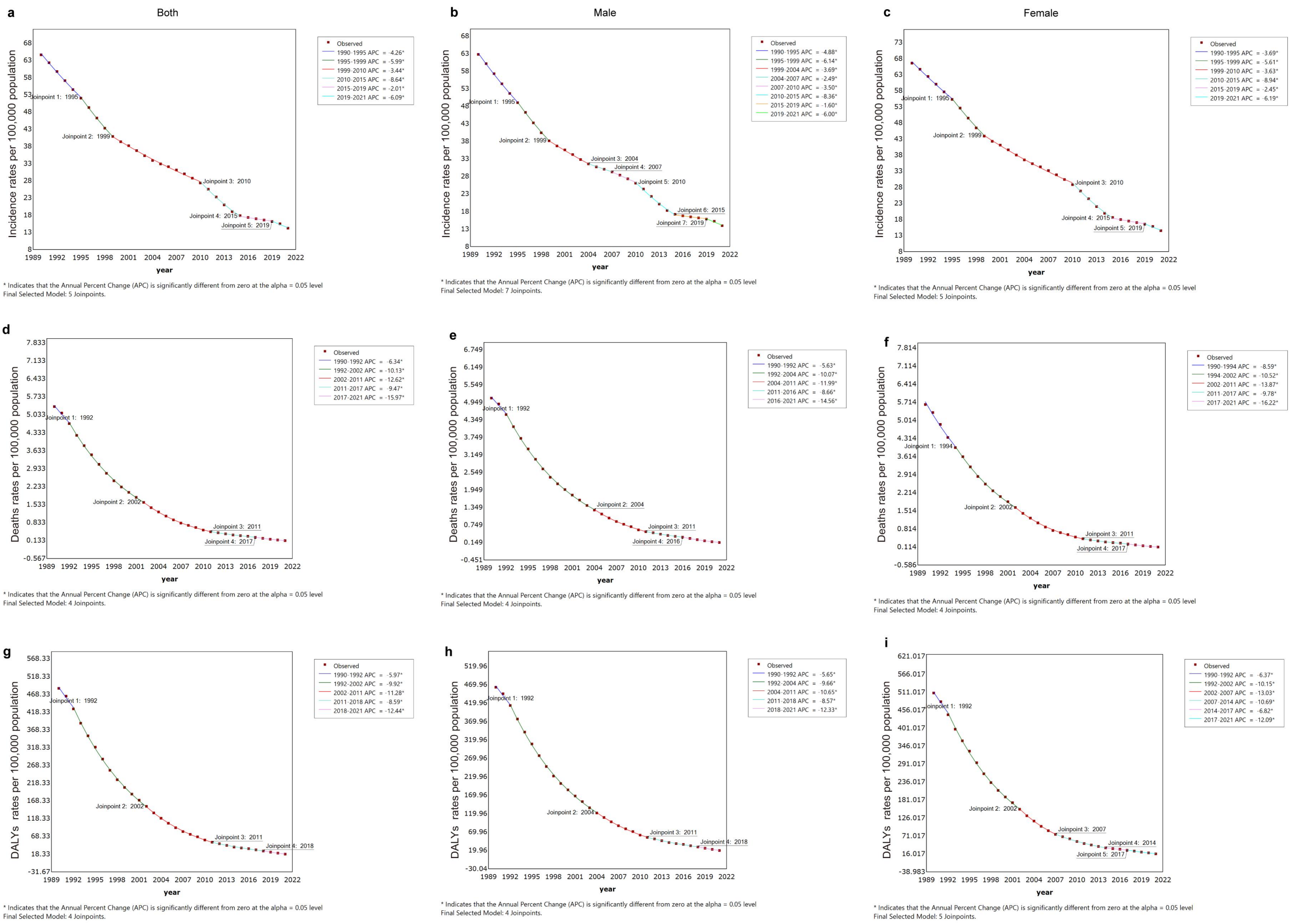

S1 Fig. Joinpoint regression results for incidence rates, death rates, and DALYs rates in age group 0-19 years, stratified by gender. a-c shows joinpoint results of incidence rates, d-f shows joinpoint results of death rates, and g-i shows joinpoint results of DALYs rates.
