# Supplementary material for: Long-term trends and future projections of the burden of tuberculosis among children and adolescents in China
Source: PLoS One. 2025 Jul 17;20(7):e0328255. doi: 10.1371/journal.pone.0328255 (PMC12270101; doi:10.1371/journal.pone.0328255)
Supplement: S2 Fig — a-d shows joinpoint results of male incidence rates in different age groups, and e-h shows joinpoint results of female incidence rates. (PDF) [file pone.0328255.s002.pdf]

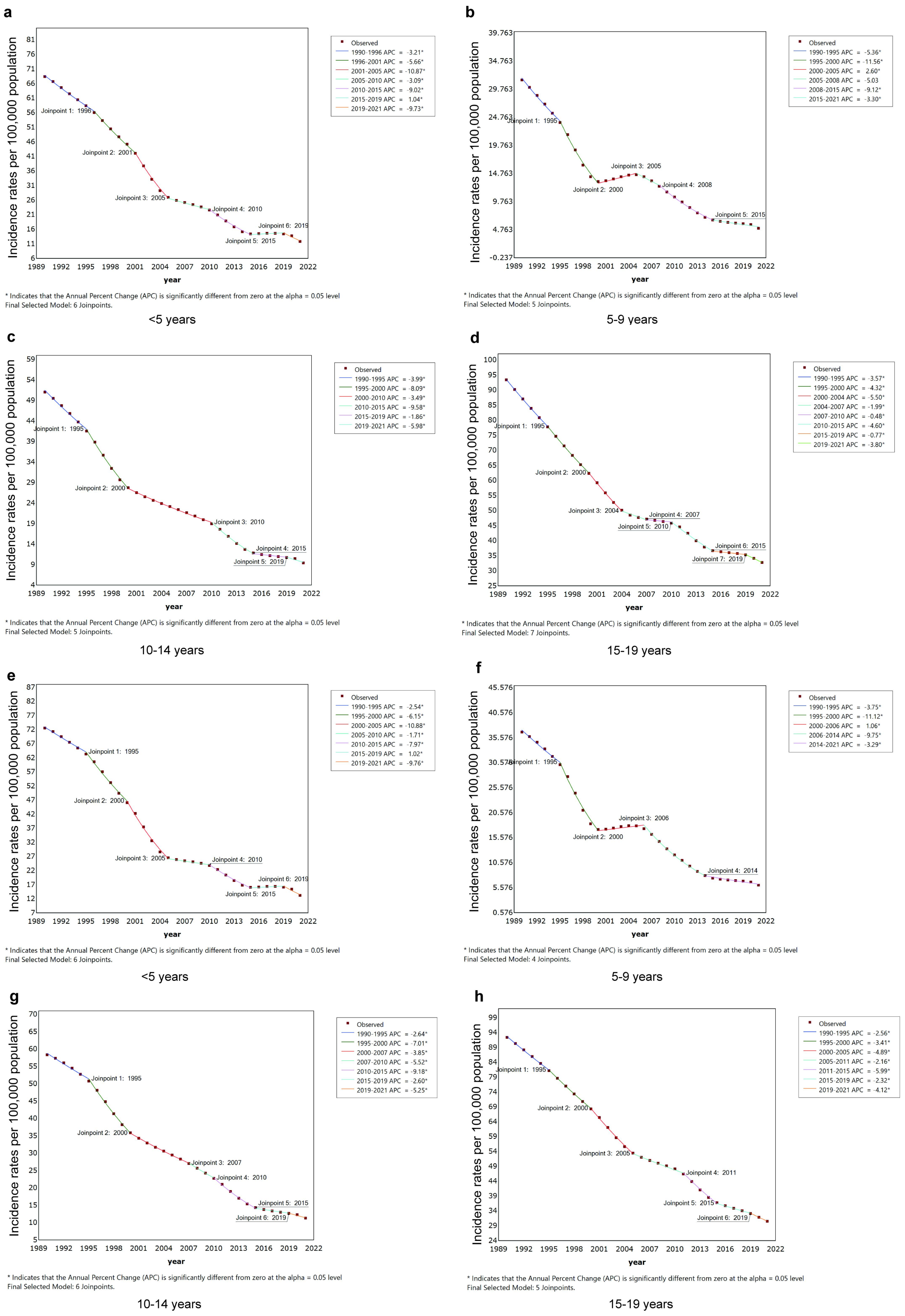


**S2 Fig. Joinpoint regression results for incidence rates across different age groups and genders.** a-d shows joinpoint results of male incidence rates in different age groups, and e-h shows joinpoint results of female incidence rates.
