# Supplementary material for: Long-term trends and future projections of the burden of tuberculosis among children and adolescents in China
Source: PLoS One. 2025 Jul 17;20(7):e0328255. doi: 10.1371/journal.pone.0328255 (PMC12270101; doi:10.1371/journal.pone.0328255)
Supplement: S1 Table — (PDF) [file pone.0328255.s005.pdf]

S1 Table. Gender-specific and age-specific incidence rates and their average annual percentage changes (AAPC) from 1990 to 2021 in China.

Gender	Age	1990 incidence rates per 100,000 population	2021 incidence rates per 100,000 population	AAPC%(1990-2021)	P
Both	<5 years	70.38 (52.6,90.42)	12.51 (9.87,15.77)	-5.39(-5.50,-5.28)	<0.001
	5-9 years	33.97 (21.2,50.33)	5.53 (3.63,8.18)	-5.57(-5.97,-5.16)	<0.001
	10-14 years	54.62 (33.85,80.12)	10.32 (6.37,15.56)	-5.22(-5.41,-5.03)	<0.001
	15-19 years	92.93 (54.24,137.33)	31.75 (20.64,45.39)	-3.41(-3.48,-3.34)	<0.001
Male	<5 years	68.41 (51.88,87.65)	11.86 (9.35,14.93)	-5.46(-5.55,-5.37)	<0.001
	5-9 years	31.42 (19.94,46.62)	5.03 (3.28,7.43)	-5.60(-6.29,-4.91)	<0.001
	10-14 years	51.07 (32.12,74.59)	9.44 (5.78,14.21)	-5.28(-5.43,-5.13)	<0.001
	15-19 years	93.45 (55.68,135.25)	32.78 (21.05,46.64)	-3.32(-3.40,-3.24)	<0.001
Female	<5 years	72.61 (54.36,93.91)	13.26 (10.44,16.78)	-5.30(-5.46,-5.14)	<0.001
	5-9 years	36.73 (23.03,54.3)	6.09 (4.02,9.11)	-5.56(-5.91,-5.21)	<0.001
	10-14 years	58.4 (35.83,86.94)	11.33 (7.01,17.02)	-5.14(-5.29,-5.00)	<0.001
	15-19 years	92.38 (52.7,139.11)	30.56 (20.19,44.26)	-3.52(-3.56,-3.47)	<0.001

AAPC=Annualised rate of change in tuberculosis incidence; GBD=Global Burden of Diseases, Injuries, and Risk Factors Study.
