# Supplementary material for: Long-term trends and future projections of the burden of tuberculosis among children and adolescents in China
Source: PLoS One. 2025 Jul 17;20(7):e0328255. doi: 10.1371/journal.pone.0328255 (PMC12270101; doi:10.1371/journal.pone.0328255)
Supplement: S2 Table — (PDF) [file pone.0328255.s006.pdf]

S2 Table. Gender-specific and age-specific death rates and their average annual percentage changes (AAPC) from 1990 to 2021 in China.

Gender	Age	1990 death rates per 100,000 population	2021 death rates per 100,000 population	AAPC%(1990-2021)	P
Both	<5 years	15.65 (13.07,18.48)	0.27 (0.21,0.34)	-12.37(-12.67,-12.08)	<0.001
	5-9 years	1.63 (1.36,1.92)	0.05 (0.04,0.06)	-10.64(-11.00,-10.29)	<0.001
	10-14 years	1.46 (1.23,1.7)	0.06 (0.05,0.08)	-9.71(-10.18,-9.25)	<0.001
	15-19 years	2.47 (2.06,2.92)	0.18 (0.14,0.22)	-8.15(-8.53,-7.77)	<0.001
Male	<5 years	14.6 (11.17,18.9)	0.31 (0.23,0.43)	-11.75(-12.16,-11.34)	<0.001
	5-9 years	1.72 (1.27,2.13)	0.06 (0.05,0.08)	-10.28(-10.79,-9.77)	<0.001
	10-14 years	1.29 (0.95,1.65)	0.06 (0.05,0.09)	-9.37(-9.90,-8.84)	<0.001
	15-19 years	2.35 (1.69,3.1)	0.20 (0.14,0.28)	-7.83(-8.12,-7.54)	<0.001
Female	<5 years	16.84 (13.83,20.29)	0.22 (0.17,0.28)	-13.10(-13.30,-12.89)	<0.001
	5-9 years	1.54 (1.28,1.8)	0.04 (0.03,0.05)	-11.24(-11.99,-10.49)	<0.001
	10-14 years	1.64 (1.36,1.92)	0.06 (0.05,0.08)	-10.04(-10.52,-9.54)	<0.001
	15-19 years	2.58 (2.13,3.03)	0.16 (0.13,0.21)	-8.42(-8.91,-7.92)	<0.001

AAPC=Annualised rate of change in tuberculosis deaths; GBD=Global Burden of Diseases, Injuries, and Risk Factors Study.
