# Supplementary material for: Long-term trends and future projections of the burden of tuberculosis among children and adolescents in China
Source: PLoS One. 2025 Jul 17;20(7):e0328255. doi: 10.1371/journal.pone.0328255 (PMC12270101; doi:10.1371/journal.pone.0328255)
Supplement: S3 Table — (PDF) [file pone.0328255.s007.pdf]

S3 Table. Gender-specific and age-specific DALYs rates and their average annual percentage changes (AAPC) from 1990 to 2021 in China.

Gender	Age	1990 DALYs rates per 100,000 population	2021 DALYs rates per 100,000 population	AAPC%(1990-2021)	P
Both	<5 years	1440.95 (1210.53,1701.25)	34.88 (28.31,43.22)	-11.34(-11.50,-11.19)	<0.001
	5-9 years	155.13 (130.03,181.84)	7.81 (5.95,10.26)	-9.05(-9.44,-8.66)	<0.001
	10-14 years	118.91 (99.83,138.38)	5.93 (4.93,7.03)	-9.26(-9.65,-8.88)	<0.001
	15-19 years	209.28 (175.87,247.52)	28.92 (21.55,38.46)	-6.20(-6.50,-5.89)	<0.001
Male	<5 years	1344.97 (1037.48,1732.3)	37.99 (30.06,48.33)	-10.93(-11.11,-10.75)	<0.001
	5-9 years	158.58 (121.32,193.81)	8.55 (6.45,11.31)	-9.01(-9.75,-8.28)	<0.001
	10-14 years	104.57 (77.38,131.86)	5.90 (4.62,7.71)	-8.88(-9.35,-8.40)	<0.001
	15-19 years	204.36 (152.95,263.67)	31.87 (22.94,44.02)	-5.82(-6.11,-5.53)	<0.001
Female	<5 years	1549.2 (1270.82,1859.12)	31.28 (24.19,38.24)	-11.83(-12.06,-11.60)	<0.001
	5-9 years	151.39 (125.94,179.52)	6.98 (5.14,9.45)	-9.42(-10.28,-8.54)	<0.001
	10-14 years	134.19 (112.14,156.39)	5.97 (4.73,7.36)	-9.60(-10.02,-9.18)	<0.001
	15-19 years	214.48 (181.34,251.22)	25.5 (18.61,34.17)	-6.60(-7.19,-5.99)	<0.001

DALYs = disability-adjusted life years; AAPC=Annualised rate of change in tuberculosis DALYs; Data in parentheses are 95% uncertainty.
