# Supplementary material for: Long-term trends and future projections of the burden of tuberculosis among children and adolescents in China
Source: PLoS One. 2025 Jul 17;20(7):e0328255. doi: 10.1371/journal.pone.0328255 (PMC12270101; doi:10.1371/journal.pone.0328255)
Supplement: S4 Table — (PDF) [file pone.0328255.s008.pdf]

S4 Table. Predicted values of incidence,deaths and DALY rates in children and adolescents of different ages and genders during 2022-2031.

Gender	Age	Year	Incidence Rate	Lower 95% CI	Higher 95% CI	Deaths Rates	Lower 95% CI	Higher 95% CI	DALYs Rates	Lower 95% CI	Higher 95% CI
Male	<5	2022	9.29	8.96	9.63	0.27	0.25	0.28	32.62	31.39	33.89
		2023	7.27	6.49	8.15	0.23	0.20	0.25	28.01	25.70	30.52
		2024	6.13	4.97	7.56	0.19	0.16	0.23	24.05	20.83	27.76
		2025	5.72	4.23	7.74	0.16	0.13	0.21	20.65	16.73	25.48
		2026	5.81	4.00	8.43	0.14	0.10	0.20	17.73	13.34	23.57
		2027	6.10	4.03	9.25	0.12	0.08	0.19	15.23	10.56	21.96
		2028	6.29	4.06	9.76	0.10	0.06	0.18	13.07	8.30	20.59
		2029	6.14	3.91	9.64	0.09	0.04	0.17	11.23	6.49	19.42
		2030	5.65	3.57	8.94	0.07	0.03	0.16	9.64	5.04	18.43
		2031	4.98	3.12	7.97	0.06	0.03	0.16	8.28	3.90	17.58
	5-9	2022	4.18	4.01	4.36	0.05	0.05	0.06	7.61	7.09	8.17
		2023	3.44	3.07	3.85	0.05	0.04	0.05	6.91	6.08	7.85
		2024	2.91	2.39	3.56	0.04	0.03	0.05	6.05	5.08	7.20
		2025	2.61	1.97	3.47	0.03	0.03	0.04	5.33	4.20	6.76
		2026	2.48	1.74	3.54	0.03	0.02	0.04	4.78	3.49	6.54
		2027	2.46	1.64	3.68	0.03	0.02	0.04	4.24	2.87	6.24
		2028	2.47	1.60	3.82	0.02	0.02	0.03	3.74	2.34	5.97
		2029	2.46	1.56	3.87	0.02	0.01	0.03	3.33	1.91	5.81
		2030	2.38	1.49	3.79	0.02	0.01	0.03	2.96	1.54	5.66
		2031	2.23	1.39	3.59	0.02	0.01	0.03	2.62	1.24	5.51
	10-14	2022	8.09	7.87	8.32	0.06	0.05	0.06	5.30	5.08	5.52
		2023	6.89	6.40	7.41	0.05	0.05	0.06	4.76	4.43	5.11
		2024	6.05	5.33	6.88	0.05	0.04	0.05	4.28	3.85	4.75
		2025	5.61	4.69	6.71	0.04	0.03	0.05	3.84	3.34	4.42
		2026	5.47	4.41	6.80	0.04	0.03	0.05	3.45	2.88	4.13
		2027	5.50	4.33	7.00	0.03	0.02	0.04	3.10	2.48	3.87
		2028	5.55	4.31	7.13	0.03	0.02	0.04	2.78	2.14	3.63
		2029	5.46	4.22	7.05	0.03	0.02	0.04	2.50	1.83	3.42
		2030	5.18	4.00	6.71	0.02	0.01	0.04	2.25	1.57	3.22
		2031	4.75	3.66	6.18	0.02	0.01	0.04	2.02	1.34	3.05
	15-19	2022	31.36	30.99	31.74	0.18	0.17	0.19	29.93	28.97	30.93
		2023	30.03	29.10	30.99	0.17	0.15	0.18	28.16	26.47	29.95
		2024	28.85	27.31	30.48	0.15	0.14	0.17	26.51	24.26	28.97
		2025	27.85	25.78	30.09	0.14	0.12	0.16	24.97	22.29	27.98
		2026	26.99	24.54	29.69	0.13	0.11	0.15	23.53	20.53	26.97
		2027	26.21	23.53	29.21	0.12	0.10	0.14	22.18	18.96	25.94
		2028	25.46	22.66	28.60	0.11	0.09	0.13	20.90	17.54	24.92
		2029	24.68	21.87	27.86	0.10	0.09	0.12	19.71	16.25	23.90
		2030	23.88	21.08	27.04	0.09	0.08	0.12	18.58	15.07	22.90

		2031	23.05	20.30	26.18	0.09	0.07	0.11	17.51	13.99	21.91
Female	<5	2022	10.73	10.33	11.15	0.19	0.18	0.20	26.58	25.45	27.76
		2023	8.69	7.84	9.64	0.16	0.14	0.18	22.59	20.50	24.89
		2024	7.43	6.19	8.92	0.13	0.10	0.17	19.20	16.32	22.58
		2025	6.89	5.32	8.91	0.11	0.08	0.16	16.31	12.86	20.69
		2026	6.89	5.02	9.45	0.09	0.06	0.16	13.86	10.05	19.12
		2027	7.19	5.05	10.24	0.08	0.04	0.16	11.78	7.79	17.82
		2028	7.52	5.18	10.91	0.07	0.03	0.16	10.01	5.99	16.72
		2029	7.57	5.18	11.08	0.06	0.02	0.16	8.51	4.58	15.80
		2030	7.21	4.91	10.58	0.05	0.01	0.16	7.23	3.48	15.03
		2031	6.50	4.41	9.58	0.04	0.01	0.17	6.14	2.62	14.39
	5-9	2022	5.14	4.97	5.31	0.03	0.03	0.03	6.03	5.56	6.55
		2023	4.28	3.85	4.75	0.03	0.02	0.03	5.30	4.53	6.21
		2024	3.69	3.03	4.48	0.02	0.02	0.03	4.71	3.74	5.92
		2025	3.35	2.52	4.45	0.02	0.01	0.03	4.21	3.13	5.66
		2026	3.19	2.24	4.56	0.02	0.01	0.03	3.78	2.64	5.41
		2027	3.13	2.08	4.70	0.02	0.01	0.03	3.40	2.24	5.15
		2028	3.07	1.97	4.79	0.01	0.01	0.03	3.07	1.92	4.90
		2029	2.97	1.86	4.75	0.01	0.00	0.03	2.77	1.65	4.64
		2030	2.82	1.73	4.58	0.01	0.00	0.03	2.50	1.42	4.38
		2031	2.62	1.58	4.34	0.01	0.00	0.03	2.26	1.23	4.13
	10-14	2022	10.06	9.86	10.26	0.06	0.05	0.06	5.32	5.07	5.59
		2023	8.81	8.35	9.30	0.05	0.05	0.06	4.75	4.35	5.18
		2024	7.80	7.08	8.60	0.04	0.04	0.05	4.23	3.72	4.81
		2025	7.11	6.17	8.19	0.04	0.03	0.05	3.77	3.17	4.49
		2026	6.72	5.62	8.04	0.04	0.03	0.05	3.36	2.69	4.21
		2027	6.55	5.32	8.05	0.03	0.02	0.04	3.00	2.27	3.96
		2028	6.49	5.19	8.12	0.03	0.02	0.04	2.67	1.91	3.74
		2029	6.43	5.10	8.12	0.02	0.02	0.04	2.38	1.60	3.54
		2030	6.29	4.96	7.97	0.02	0.01	0.04	2.13	1.34	3.37
		2031	6.01	4.72	7.63	0.02	0.01	0.04	1.90	1.12	3.21
	15-19	2022	29.41	29.19	29.63	0.15	0.14	0.15	23.30	22.33	24.30
		2023	28.41	27.73	29.12	0.13	0.12	0.15	21.73	19.80	23.86
		2024	27.52	26.30	28.80	0.12	0.10	0.14	20.28	17.90	22.97
		2025	26.66	24.97	28.47	0.11	0.09	0.13	18.92	16.29	21.98
		2026	25.80	23.78	27.99	0.10	0.08	0.13	17.65	14.87	20.95
		2027	24.92	22.72	27.32	0.09	0.07	0.12	16.47	13.61	19.92
		2028	24.03	21.76	26.53	0.08	0.06	0.11	15.36	12.48	18.91
		2029	23.15	20.88	25.68	0.08	0.06	0.11	14.33	11.46	17.93
		2030	22.32	20.04	24.84	0.07	0.05	0.10	13.37	10.54	16.98
		2031	21.52	19.26	24.05	0.06	0.04	0.09	12.48	9.69	16.06
	<5	2022	9.79	9.45	10.16	0.23	0.22	0.24	29.82	28.65	31.03
		2023	7.65	6.82	8.57	0.20	0.17	0.22	25.49	23.32	27.87

Both		2024	6.41	5.18	7.93	0.17	0.13	0.21	21.79	18.78	25.29
		2025	5.93	4.36	8.08	0.14	0.10	0.20	18.63	14.98	23.17
		2026	5.98	4.08	8.78	0.12	0.08	0.19	15.93	11.86	21.40
		2027	6.28	4.08	9.66	0.10	0.06	0.19	13.62	9.32	19.91
		2028	6.50	4.11	10.27	0.09	0.04	0.19	11.64	7.27	18.65
		2029	6.40	4.00	10.26	0.07	0.03	0.19	9.96	5.64	17.58
		2030	5.95	3.68	9.62	0.06	0.02	0.19	8.51	4.35	16.67
		2031	5.29	3.24	8.65	0.05	0.02	0.19	7.28	3.33	15.89
	5-9	2022	4.69	4.51	4.87	0.04	0.04	0.04	6.64	6.22	7.09
		2023	3.93	3.54	4.35	0.04	0.03	0.04	5.92	5.11	6.84
		2024	3.38	2.82	4.04	0.03	0.03	0.04	5.27	4.27	6.51
		2025	3.05	2.36	3.94	0.03	0.02	0.03	4.70	3.57	6.18
		2026	2.90	2.10	3.99	0.02	0.02	0.03	4.18	2.98	5.88
		2027	2.86	1.99	4.11	0.02	0.01	0.03	3.73	2.48	5.60
		2028	2.86	1.93	4.22	0.02	0.01	0.03	3.32	2.07	5.34
		2029	2.83	1.89	4.25	0.02	0.01	0.03	2.96	1.72	5.10
		2030	2.73	1.81	4.14	0.01	0.01	0.02	2.64	1.42	4.89
		2031	2.56	1.68	3.91	0.01	0.01	0.02	2.35	1.17	4.70
	10-14	2022	9.00	8.80	9.22	0.06	0.05	0.06	5.31	5.09	5.54
		2023	7.78	7.30	8.28	0.05	0.05	0.06	4.76	4.41	5.13
		2024	6.86	6.14	7.67	0.05	0.04	0.05	4.26	3.80	4.77
		2025	6.32	5.40	7.39	0.04	0.03	0.05	3.81	3.27	4.45
		2026	6.08	5.01	7.38	0.04	0.03	0.05	3.42	2.80	4.16
		2027	6.04	4.86	7.50	0.03	0.02	0.04	3.06	2.39	3.91
		2028	6.05	4.81	7.61	0.03	0.02	0.04	2.74	2.04	3.68
		2029	5.98	4.73	7.57	0.03	0.02	0.04	2.45	1.73	3.48
		2030	5.75	4.53	7.30	0.02	0.01	0.04	2.20	1.46	3.30
		2031	5.37	4.22	6.83	0.02	0.01	0.04	1.97	1.24	3.13
	15-19	2022	30.72	30.44	31.00	0.16	0.16	0.17	26.95	26.09	27.84
		2023	29.86	29.00	30.75	0.15	0.14	0.16	25.28	23.62	27.07
		2024	29.03	27.53	30.62	0.14	0.12	0.16	23.72	21.66	25.98
		2025	28.16	26.13	30.35	0.13	0.11	0.15	22.25	19.96	24.81
		2026	27.23	24.85	29.84	0.12	0.10	0.14	20.88	18.44	23.64
		2027	26.28	23.71	29.13	0.11	0.09	0.13	19.59	17.06	22.48
		2028	25.35	22.68	28.32	0.10	0.08	0.12	18.38	15.81	21.36
		2029	24.46	21.75	27.50	0.09	0.07	0.11	17.24	14.66	20.27
		2030	23.62	20.89	26.71	0.08	0.07	0.10	16.17	13.61	19.22
		2031	22.83	20.08	25.97	0.08	0.06	0.10	15.17	12.64	18.22
