# Supplementary material for: Long-term trends and future projections of the burden of tuberculosis among children and adolescents in China
Source: PLoS One. 2025 Jul 17;20(7):e0328255. doi: 10.1371/journal.pone.0328255 (PMC12270101; doi:10.1371/journal.pone.0328255)
Supplement: S5 Table — (PDF) [file pone.0328255.s009.pdf]

S5 Table. AIC values for suggested ARIMA models and Ljung-Box test results for incidence, deaths and DALYs rates of different age groups.

	Age	Gender	ARIMA Model	AIC	Ljung-Box	
					χ^2	P
Incidence	<5	Male	(2,1,1)	-148.78	7.3516	0.69
		Female	(2,1,0)	-147.62	8.9902	0.53
	5-9	Male	(2,1,0)	-141.97	5.5586	0.85
		Female	(2,1,1)	-154.54	9.3134	0.50
	10-14	Male	(2,1,0)	-168.11	6.835	0.74
		Female	(2,1,0)	-189.21	8.8718	0.54
	15-19	Male	(2,1,0)	-221.45	3.6036	0.96
		Female	(2,1,1)	-248.21	4.0062	0.95
Deaths	<5	Male	(0,2,0)	-136.64	8.3627	0.59
		Female	(0,2,1)	-132.12	7.6574	0.66
	5-9	Male	(3,2,0)	-102.69	7.0533	0.72
		Female	(0,2,1)	-93.10	11.361	0.33
	10-14	Male	(0,2,1)	-135.34	5.8056	0.83
		Female	(0,2,1)	-126.79	8.6306	0.57
	15-19	Male	(0,1,1)	-141.90	13.695	0.19
		Female	(1,1,0)	-123.65	13.467	0.20
DALYs	<5	Male	(0,2,0)	-149.13	13.362	0.20
		Female	(0,2,0)	-141.76	23.357	0.01
	5-9	Male	(2,2,0)	-109.44	10.392	0.41
		Female	(1,1,0)	-104.35	5.2071	0.88
	10-14	Male	(0,2,1)	-143.47	5.6927	0.84
		Female	(0,2,1)	-132.91	10.142	0.43
	15-19	Male	(1,1,0)	-161.56	15.668	0.11
		Female	(0,1,1)	-143.52	12.165	0.27
